# Supplementary material for: Probabilistic variable-length segmentation of protein sequences for discriminative motif discovery (DiMotif) and sequence embedding (ProtVecX)
Source: Sci Rep. 2019 Mar 5;9:3577. doi: 10.1038/s41598-019-38746-w (PMC6401088; doi:10.1038/s41598-019-38746-w)
Supplement: Supplementary file 1 — SUPPLEMENTARY MATERIAL [file 41598_2019_38746_MOESM1_ESM.pdf]

Supplementary material

Probabilistic variable-length segmentation of protein sequences for discriminative motif discovery (DiMotif) and sequence embedding (ProtVecX)

Ehsaneddin Asgari[1,2], Alice McHardy[2], and Mohammad R.K. Mofrad[1,3,*]

[1]Molecular Cell Biomechanics Laboratory, Departments of Bioengineering and Mechanical Engineering, University of California, Berkeley, CA, 94720, USA
[2]Computational Biology of Infection Research, Helmholtz Centre for Infection Research, Brunswick 38124, Germany
[3]Molecular Biophysics and Integrated Bioimaging, Lawrence Berkeley National Lab, Berkeley, CA 94720, USA
[*]mofrad@berkeley.edu

Introduction to embedding representations

In order to discuss a complex concept with an audience unfamiliar with the topic, we model or represent the concept within a framework that is understandable for the audience. The same logic applies to present a natural language text to a computer. Computers are experts in dealing with numerical values, vectors, and matrices. Thus, the first step in processing text is to vectorize it for computers. Words are the input units of almost all natural language processing (NLP) tasks. Therefore, to utilize machines for language processing we need to find proper vector representations of words that are interpretable by machines. We expect such representations to preserve some indications of similarity and dissimilarity between words. For instance, when we search a phrase in a search engine we expect the machine to consider words 'formula' and 'equation' to be similar and consider them dissimilar to an irrelevant word like 'cuisine'. Thus, we should attribute similar vector representations to the words 'formula' and 'equation', dissimilar to the vector representation of 'cuisine'. As a reminder vector similarity can be calculated using operations in linear algebra (e.g. dot product, Euclidian distance, and cosine similarity). Of course, semantic similarity is not the only consideration exist in NLP tasks. As an example, part-of-speech tagging is one of the routine NLP tasks, where the goal is to label words with their syntactic part-of-speech (e.g. verb, adverb, etc.). Presumably, when we want to perform part-of-speech tagging we desire a vector representation incorporating syntactic similarities.

Continuous vector representations known as word vectors have recently become popular in NLP as an efficient approach to represent semantic/syntactic units [1]. Word vectors are trained in the course of training neural network based language models from large amounts of textual data (words and their contexts). To be more precise, word representations are the outputs of the last hidden layers in neural networks trained for prediction of the context of a given word, which is analogous to language modeling. Thus, word vectors are supposed to encode the most relevant features to language modeling by observing various samples. In such a representation similar words have closer vectors, where similarity is defined in terms of both syntax and semantics. By training word vectors over large corpora of natural languages, interesting patterns have been observed. Words with similar vector representations display various types of similarity. For instance, $King - Man + Woman$ is the closest vector to that of the word *Queen* (an instance of semantic regularities) and $quick - quickly \approx slow - slowly$ (an instance of syntactic regularities) [1]. Word vectors are used as general-purpose data representation methods in many natural language processing applications including part-of-speech tagging, machine translation, and information retrieval [2].

Protein-vectors (ProtVec) adopt such representation for biological sequences [3]. ProtVec is word vector representations for segments (k-mers) of biological sequences, which can be utilized as the representation method for a wide array of tasks in bioinformatics. For training word vectors in NLP, a

large corpus should be used to ensure that a sufficient number of contexts have been observed for all words. Similarly, for protein sequences, Swiss-Prot as a rich protein database, which consists of $\approx 600K$ manually annotated and reviewed sequences. For training of protein vectors, we break the sequences into subsequences. The simplest and most common technique in bioinformatics to study sequences involves fixed-length overlapping k-mers. However, instead of using k-mers directly in feature extraction, we utilize k-mer modeling for training a general purpose vector representation of sequences. This so-called embedding model needs to be trained only once and may then be adopted in feature extraction part of specific problems. For more details on ProtVec training please refer to [3].

References

1. Mikolov, T., Sutskever, I., Chen, K., Corrado, G. S. & Dean, J. Distributed representations of words and phrases and their compositionality. In *Advances in Neural Information Processing Systems*, 3111–3119 (2013).

2. Levy, O. & Goldberg, Y. Neural word embedding as implicit matrix factorization. In *Advances in Neural Information Processing systems*, 2177–2185 (2014).

3. Asgari, E. & Mofrad, M. R. Continuous distributed representation of biological sequences for deep proteomics and genomics. *PloS One* **10**, e0141287 (2015).
